# Seasonal-Spatial Distribution Variations and Predictions of *Loliolus beka* and *Loliolus uyii* in the East China Sea Region: Implications from Climate Change Scenarios

**DOI:** 10.3390/ani14142070

**Published:** 2024-07-15

**Authors:** Min Xu, Wangjue Feng, Zunlei Liu, Zhiguo Li, Xiaojing Song, Hui Zhang, Chongliang Zhang, Linlin Yang

**Affiliations:** 1 Key Laboratory of East China Sea Fishery Resources Exploitation, Ministry of Agriculture and Rural Affairs, Shanghai 200090, China; xumin@ecsf.ac.cn (M.X.);; 2 East China Sea Fisheries Research Institute, Chinese Academy of Fishery Sciences, Shanghai 200090, China; 3 Fisheries College, Ocean University of China, Qingdao 266003, China; 4 Xiangshan County Fisheries Bureau, Ningbo 315700, China

**Keywords:** climate warming, species distribution model, *Loliolus uyii*, *Loliolus beka*, sdmTMB, SSP1-2.6, SSP2-4.5, SSP5-8.5, Cephalopoda, Loliginidae

## Abstract

**Simple Summary:**

*Loliolus* spp., such as *Loliolus beka* (Sasaki, 1929) and *Loliolus uyii* (Wakiya and Ishikawa, 1921), represent a dominant fishing bycatch in the East China Sea. However, their seasonal–spatial distribution characteristics are unclear. Here, the generalized linear mixed-effects model sdmTMB was used to evaluate ways of handling merged distribution data for *L. beka* and *L. uyii* to predict the variation in their spatial distributions under different climate change scenarios. Model predictions were validated using cross-validation, while model performance was assessed using *T*-tests. We speculated that the range and density of both species will vary over time, with some areas in the mid-East China Sea becoming potential habitats. In addition, we assumed that individuals of both species in the northern survey area might migrate to the northern Yellow Sea and Bohai Sea to spawn, whereas those in the central and southern Yellow Sea might migrate to coastal areas. During autumn, newborn larvae in the northern survey area would migrate to the southern Yellow Sea, whereas those in the central and southern areas would gradually migrate to offshore areas, resulting in a distribution ranging from coastal sea areas to the central survey area. Thus, this study provides novel insights into modeling habitat distributions for data-limited species and theoretical support for adapting fisheries resource management to climate change.

**Abstract:**

Global climate change profoundly impacts the East China Sea ecosystem and poses a major challenge to fishery management in this region. In addition, closely related species with low catches are often not distinguished in fishery production and relevant data are commonly merged in statistics and fishing logbooks, making it challenging to accurately predict their habitat distribution range. Here, merged fisheries-independent data of the closely related squid *Loliolus beka* (Sasaki, 1929) and *Loliolus uyii* (Wakiya and Ishikawa, 1921) were used to explore the construction and prediction performance of species distribution models. Data in 2018 to 2019 from the southern Yellow and East China Seas were used to identify the seasonal–spatial distribution characteristics of both species, revealing a boundary line at 29.00° N for *L. uyii* during the autumn, with the highest average individual weight occurring during the summer, with both larvae and juveniles occurring during the autumn. Thus, the life history of *L. uyii* can be divided into winter–spring nursery and summer–autumn spawning periods. *L. beka* showed a preference for inshore areas (15–60 m) during the summer and offshore areas (32.00–78.00 m) during the winter. High-value areas of both species included inshore areas of the southern Yellow and mid-East China Seas during the autumn, enlarging during the spring to include central areas of the survey region, before significantly decreasing during the summer. Therefore, this study provides both a novel perspective for modeling biological habitat distribution with limited data and a scientific basis for the adjustment of fishery resource management and conservation measures in the context of climate change.

## 1. Introduction

Squid from the *Loliolus* genus, including the closely related *Loliolus beka* (Sasaki, 1929) and *Loliolus uyii* (Wakiya and Ishikawa, 1921) (Mollusca, Cephalopoda, Teuthoidea, Myopsida, Loliginidae), have weak swimming ability and move with the tide, mainly feeding on crustaceans and larvae of the economically important small yellow croaker *Larimichthys polyactis* [1], with a lifespan of <1 year. They inhabit the tropical and temperate waters of the North Pacific, including the seas around China, southern Japan, the Korean peninsula, and Indonesia [2]. Reproductive populations of *L. beka* occur throughout the year, peaking from August to September and reaching sexual maturity the following year [3]. *Loliolus* with a body length of 34–77 mm are often caught as bycatch in anchored stow nets, otter trawls and shrimp beam trawls [2]. The *L. beka* catch is thought to represent 50% of the total catch of *Loliolus* spp. in the East China Sea [3]. *L. beka* is the dominant species in the Minnan fishing grounds [4] and Zhejiang coastal area (37.00°–38.50° N 118.45°–120.50° E) [5]. However, given their morphological similarities, the two species are not always accurately identified and, thus, further insights into the current resource status and life-history characteristics of both species are warranted.

In addition, global climate changes have become a focus of the scientific community. The ‘*Blue Book on Climate Change 2023*’ issued by the China Meteorological Administration, highlights China as experiencing significant impacts of global climate change, and the alterations of ocean variables, such as increasing sea surface temperature (SST), might have significant impacts on marine ecosystems [6], also including the variations of the growth, reproductive patterns, spatial distribution range of marine organisms, and even might lead to the changes in migration pattern of some species [7]. For example, a strong positive relationship was found between *Loliolus* squid catch and autumn SST, which implied that a warmer environment might accelerate the growth of individual colder *Loliolus* squid, especially during the larval and juvenile stages, thus increasing recruitment success and subsequent catch [8]. The sudden cooling of the environment that occurred in 1977 and 1981 might have suppressed the hatching process and reduced the growth and survival of the recruitment population [9], thus resulting in poor fishery performance. Thus, in the scale of global climate change, it is vital to study the seasonal–spatial distribution characteristics of and predict future trends in the availability of fishery resources to both support the protection and sustainable utilization of cephalopod resources. Previous studies have not adequately addressed the combined effects of seasonal and spatial variations on *Loliolus* distributions under different climate change scenarios.

Moreover, the 1976–1977 regime shift in the North Pacific was commonly recognized as a shift from a ‘negative’ to a ‘positive’ phase in the Pacific Decadal Oscillation (PDO), resulting in substantial environmental change [10]. There were significant negative effects of the Asian Monsoon Index (AMI) on common Chinese cuttlefish because of the associated increased winter water temperature. Capture data also reveal a dramatic expansion in the total cephalopod biomass over the past two decades, which could be partly explained by the increase in the Loliolus, which have benefited from the continual warming of their environment over this time [11]. Shang et al. (2002) and Hong et al. (2005) reported that the catch of warm water pelagic fish increased in 1997, coinciding with a strong El Niño event; by contrast, some species, such as *Loliolus* spp., tend to move further north or into deeper water in response to global warming [12,13,14]. Pang et al. (2018) concluded that the China Sea is one of the most rapidly warming large marine ecosystems in the world [8]; other authors suggested that coastal cephalopods, in contrast to the concurrent serious declines in the abundance of fish species, display a positive response to environmental changes, resulting from their flexible life histories and ability to adapt quickly to changing environmental conditions [15].

Finally, it is challenging to accurately identify and predict the habitat distribution range of a species owing to the difficulties of monitoring. Species distribution models (SDMs) are used to predict the current and future spatial distribution of species, including the impacts of temporal variations and impacts on model performance from data quantity, errors, and distribution, via analyzing the relationship between species presence (and abundance) and environmental variables [16,17,18,19]. Owing to the similarities of morphological characteristics among different species in some taxonomic groups as well as morphological variations in the individuals of the same species, it is difficult to accurately identify the species in situ [20,21]. In this study, (1) merged fisheries-independent data relating to *L. beka* and *L. uyii* were used to explore the construction and prediction performance of SDMs. The results of models relating to the two species individually and of those based on merged data were examined to explore how the approaches used to merge the data impact the prediction of the habitat distribution of these species. A spatiotemporal generalized linear mixed effects model (GLMM), sdmTMB, was developed to manage the spatial autocorrelation structure in the species distribution, and cross-validation methods were used to assess the predictive performance of the resulting models. Additionally, (2) the future distribution dynamics of *L. beka* and *L. uyii* in the survey areas under the context of climate change were predicted via the use of shared socioeconomic path (SSP) climate data. (3) Fisheries-independent survey data from the southern Yellow Sea and East China Sea from 2018 to 2019 were also utilized to identify seasonal–spatial distributions and life-history characteristics of these two species correlated with environmental variables.

## 2. Materials and Methods

### 2.1. Data Source and In Situ Surveys

**Figure 1:**
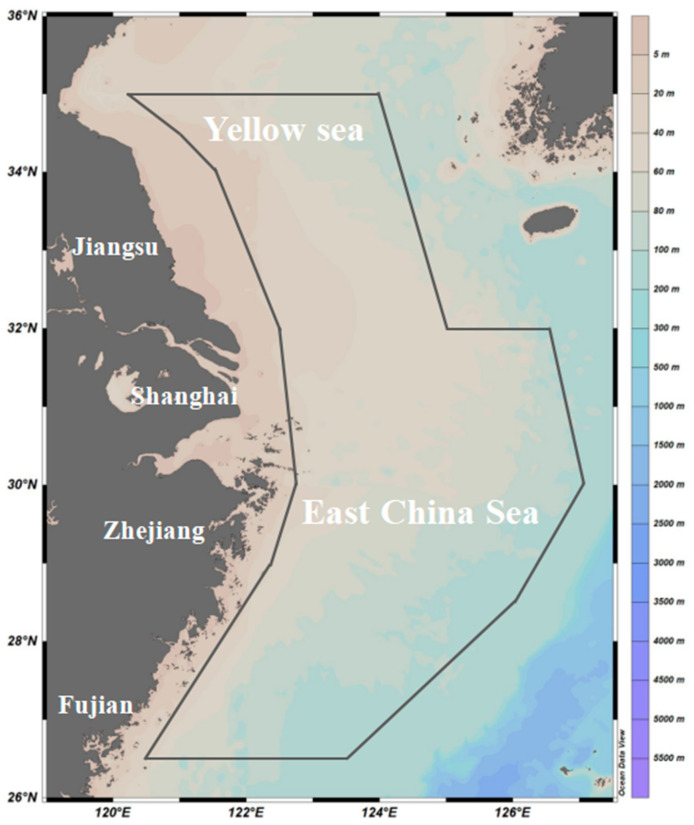
Map of the survey area (26.50° N–35.00° N 120.00° E–127.00° E) denoted by a black solid line border in the southern Yellow Sea and the East China Sea adjacent to the coastline of Jiangsu, Shanghai, Zhejiang, Fujian outside the area closed for marine trawl fisheries. The color bar denotes the depth range from 0 m to 5500 m.

The bottom trawling survey data regarding *L. beka* and *L. uyii* were collected during four consecutive seasonal cruises conducted from 2018 to 2019 in the southern Yellow Sea and East China Sea (autumn: 2–11 November 2018 with 127 sampling stations; winter: 4–27 January 2019 with 111 stations; spring: April 22–May 10, 2019 with 141 stations; and summer: 13 August–27 September 2019 with 140 stations) [22]. The surveys were conducted by research vessels (#Zhongkeyu 211 and 212) [23,24] using a trawl net with a 102-mesh mouth size, a headline of 72.24 m, a groundline of 82.44 m, and a cod end mesh size of 20 mm, and 1-h trawling time. The survey areas primarily covered between 26°30′ N and 35°00′ N latitude, and 120°00′ E to 127°00′ E longitude, with stations spaced at longitude 30 min × latitude 30 min intervals using a grid method (Figure 1). The in situ survey in all seasons was performed by adopting a snake-like pattern along the route. In the southern area below 30°00′ N latitude, the survey was performed from north to south. In the northern area above 30°00′ N latitude, the survey was conducted from south to north. Species identification of the catch was performed in the laboratory to determine the presence of the two squid species at each station. The squid were counted and weighed to the nearest 0.1 g of wet weight in the laboratory. The biomass index representing catch density per unit of time was measured using two components: biomass density (unit: g·h^−1^) and individual number density (unit: ind·h^−1^). Hydrographic data, including depth, water temperature, salinity, and dissolved oxygen (DO) concentration, were also collected at each survey station using a conductivity-temperature-depth profiler (CTD, SBE-19) (SeaBird-Scientific, Bellevue, WA, USA): sea surface temperature, salinity and dissolved oxygen were measured within 3 m below the surface (SST, SSS, and SSDO, respectively), and sea bottom temperature, salinity and dissolved oxygen (SBT, SBS, and SBDO, respectively) were measured 2 m above the seabed in water shallower than 50 m and between 2 and 4 m above the seabed in deeper water.

The following equations were used to calculate the catch per unit effort (CPUE) [22,25]:

$${\mathrm{CPUE}}_{n}=\frac{N_{i}}{t_{i}}$$

$${\mathrm{CPUE}}_{w}=\frac{W_{i}}{t_{i}}$$

where $N_{i}$ is the catch in number (ind) at i station; $W_{i}$ is the catch in weight (g) at i station; and $t_{i}$ is the trawling time (h) at i station. Additionally, we defined the average individual weight (AIW) as the ratio of the CPUE by weight (CPUE_w_) against CPUE by number (CPUE_n_) at a station.

### 2.2. Introduce and Define SSP1-2.6, SSP2-4.5 and SSP5-8.5

In addition, we downloaded mean SST and SSS data from the website Bio-ORACLE: Marine data layers for ecological modelling (https://bio-oracle.org/index.php; accessed on 11 June 2024) in choosing the conditions of time the 2050s/2090s and three SSP scenarios. SSP1-2.6 shows a sustainable development scenario that emphasizes sustainability, low resource consumption, and low carbon emissions; SSP2-4.5 shows a middle-of-the-road scenario that falls between high and low emission pathways, reflecting a more moderate socio-economic development pattern; SSP5-8.5 shows a fossil fuel-driven development scenario characterized by high carbon emissions, assuming future society heavily relying on fossil fuels to power economic growth.

### 2.3. sdmTMB Model

The SDM model was used in complex ecosystems with dynamic networks characterized by nonlinear and non-additive processes. The sdmTMB model used in this study is a spatiotemporal generalized linear mixed effects model (GLMM), which utilizes the advantages of Template Model Builder (TMB), R-INLA, and Gaussian Markov Random Fields (GMRF) so that it can deal with complex spatial autocorrelation structures, high-efficient model fitting, and flexible model configuration [26]. The structure of the sdmTMB model was described by the following equations:

$$E[y_{s,t}]=\mu_{s,t}$$

$$\mu_{s,t}=f^{-1}\left({Χ}_{s,t}^{\mathrm{main}}\beta +O_{s,t}+\alpha_{g}+{Χ}_{s,t}^{tvc}\gamma_{t}+X_{s,t}^{svc}\zeta_{s}+\omega_{s}+\epsilon_{s,t}\right)$$

where $y_{s,t}$ represents the observed value of the response variable at spatial location s and time t, and μ represents its expected value; f represents a link function, and $f^{-1}$ represents its inverse function. ${Χ}_{s,t}^{main}{,\;Χ}_{s,t}^{tvc}$, and $X_{s,t}^{svc}$ represent different design matrices, where the superscript symbol ‘main’ represents main effects; ‘tvc’ represents time-varying coefficients, and ‘svc’ represents spatially varying coefficients. The corresponding β, $\gamma_{t}$ and $\zeta_{s}$ represent fixed-effect coefficients, time-varying coefficients, and variable spatial coefficients, respectively. $\gamma_{t}$ represents a random walk process or time autocorrelation, assuming obeying normal distribution, with the average value represented by the coefficient of the previous time step $\gamma_{t-1}$ with the variance of $\sigma_{\gamma }^{2}$; $\zeta_{s}$ is modeled as a random field in which the values at each spatial position *s* are random and have spatial correlations with the values at other locations, assuming obeying multivariate normal distribution (MVN) with a mean of 0 and a covariance matrix of $\sum_{\zeta }$. $O_{s,t}$ represents the offset, that is, a covariate with a fixed coefficient of 1; and $\alpha_{g}$ represents random intercepts set by the groups, allowing the model to have different baseline levels between different groups, thus reflecting inter-group heterogeneity.

In addition, sdmTMB can deal flexibly with random effects. Spatial random effect $\omega_{s}$ (also called spatial residuals) is used to reflect spatial correlations in response variables that are not explained by covariates. Similar to the variable spatial coefficient $\zeta_{s}$, the spatial random effect $\omega_{s}$ is also modeled as a random field, and its distribution is also assumed to be an MVN with a mean of 0 and a mean square error matrix of $\sum_{\omega }$. The spatiotemporal random effect $\epsilon_{s,t}$ (also called spatiotemporal residuals) is used to capture the response variable of temporal and spatial correlations unexplained by covariates, temporal tendency, spatial effect and other random effects $\epsilon_{s,t}$. $\epsilon_{s,t}$ is also modeled as a random field, the distribution of which is also assumed to have a MVN with a mean of 0 and a covariance matrix of $\sum_{\epsilon }$. In general, the sdmTMB model can contain fixed effects, random effects, time-varying coefficients, spatially varying coefficients, and spatiotemporal random effects, among others, and can flexibly establish complex spatiotemporal models.

### 2.4. Model Construction and Variable Selection

The presence or absence of the target species at each site was treated as the response variable and the error distribution family of the sdmTMB model was set to a binomial distribution. Environmental variables and spatiotemporal factors were selected as explanatory variables; the environmental variables included were depth, sea surface temperature (SST), sea bottom temperature (SBT), sea surface salinity (SSS), and sea bottom salinity (SBS); the spatiotemporal factors were month, longitude, and latitude, and they were not modeled as fixed effects but used instead to construct random effects [27]. Specifically, the latitude and longitude of spatial coordinates in the fisheries survey data were converted into the universal transverse Mercator grid system and a spatial grid was then created to apply the stochastic partial differential equation (SPDE) method [28]. The SPDE method is based on the gauss markov random field (GMRF), and discretizes the spatial process into a GMRF. The grid cells provide the discrete structure needed to construct the GMRF, allowing the parameters of the GMRF in each cell to be defined and the spatial random effects to be characterized through the SPDE method, thus effectively handling spatial correlations and enhancing the computational efficiency and flexibility of the model [29].

For environmental factors, smoothing functions were used to handle the nonlinear relationships between explanatory variables and the response variable to enhance the predictive capability of the model. The environmental factors were screened preliminarily to ensure the ecological validity of the model and stepwise regression was then used to select the most important variables, following the Akaike information criterion (AIC) [30]. The created model procedure first involved constructing a simple model without any explanatory variables and then attempting to incorporate spatial and spatiotemporal effects to verify whether the model can fit such effects. Then the selected environmental factors were added or deleted one by one and the process was repeated until the model with the least AIC value was selected as the theoretically optimal model.

AIC was calculated by using the equation:

AIC=2k−2lnL

where k is the number of parameters and L is the likelihood function. The purpose of AIC is to find a balance point between the fitting ability of model and the complexity; the smaller the value was, the better the fit of the model to the data was while considering the complexity of those data.

### 2.5. Predictive Power

Given that the AIC cannot directly assess the predictive ability of a model on new data, cross-validation was conducted to further verify the reliability of the model and to exclude the problem of a theoretically good fit but poor predictive ability (i.e., overfitting) [31]. For the model not considering spatiotemporal effects, owing to data structure not involving temporal series, the k-fold cross-validation method was used for model validation, which divides the dataset into multiple groups according to a preset number of folds (k folds), rotating each group as the test set and the rest as the training set, to comprehensively evaluate the performance of the model. For models incorporating spatiotemporal effects, the leave future out cross-validation (LFOCV) strategy was adopted [32]. In this validation method, all data up to the *t*-th year are used as the training set to predict the situation in the t + 1 year, and so on. The sum of the logarithmic likelihood values was used as the key index for assessing the predictive ability of the model; that is, the model was used to predict for each test set and the logarithmic likelihood values were then calculated between the predicted outcomes and the actual observations. By aggregating the logarithmic likelihood values for each test set, it was possible to assess the average predictive performance of the model across different datasets, reflecting its overall predictive ability. In total, 100 cross-validations were performed for each model and the average value was taken to assess the overall predictive power of the model.

In addition, the AUC value, Matthew’s correlation coefficient (MCC), and Brier score were used to assess the consistency of the predicted results with the actual sampling distribution. The AUC value is a commonly used index to assess the performance of binary classification models [33]. A high AUC value indicates that the model can better distinguish the location of species occurrence and non-occurrence, and the predicted results are more consistent with the actual distribution. MCC [34] takes into account all true and false positive and negative cases, and is a balance measure that can remain effective even in the case of unbalanced data categories. The Brier score [35] is another evaluation criterion of prediction accuracy. A lower value indicates a higher prediction accuracy by calculating the squared average of the difference between the forecast probability and the actual outcome.

MCC was calculated using the equation:

$$MCC=\frac{TP\times TN-FP\times FN}{\sqrt{\left(TP+FP)(TP+FN)(TN+FP)(TN+FN\right)}}$$

where TP represents a true positive (i.e., the positive case of correct prediction); TN represents a true negative (i.e., the negative example of correct prediction); FP indicates a false positive (i.e., a positive example of a false prediction); and FN represents a false negative (i.e., a negative example of a wrong prediction).

Brier scores were calculated by using the following equation:

$$Brier\;Score=\frac{1}{N}\sum_{i=1}^{n}{\left(p_{i}-o_{i}\right)}^{2}$$

where N is the number of samples, $p_{i}$ is the prediction probability, and $o_{i}$ is the real result corresponding to the sample: 1 if the event occurs, and 0 if not.

Two modeling-prediction strategies were evaluated to test the effects of merging species data. First, the two squid species were modeled separately without data merging; then, the spatial distribution of each species was predicted, and these prediction results were combined to obtain the overall distribution probability of both squid species, which was called the prediction-pool method. The other approach was to merge the data first and then create a model based on the aggregated data, which was then used for prediction, called the data-pool approach. In general, the latter is often encountered in practice (such as fishing log data), whereas the former is not common but can be viewed as a simulation test conducted for comparative analysis and provides a reference for evaluating the effectiveness of the data-pool method.

### 2.6. Prediction of Habitat Spatial Distribution

The probability of occurrence of specific species at specific times and locations was estimated based on the selected optimal models. To investigate the seasonal distribution of *L. beka* and *L. uyii* throughout the survey area, the forecast area was divided into 10,201 grid points (101 × 101) in the area of 119.5° E–127.0° E 26.0° N–35.5° N according to the UTM coordinate system. The environmental variables used in the forecast were monthly mean SBT, monthly mean SBS, monthly mean SSS, and bathymetric data for November 2018, and January, May, and August 2019. The depth data were collected from GEBCO (GEBCO Compilation Group 2023), and temperature and salinity data were from the European Union Copernicus Marine service information (https://doi.org/10.48670/moi-00021; accessed on 5 May 2024).

In addition, the variation in the distribution probability of these two species in the survey area under the background of future climate change was forecast to explore the potential change trend in the resource distribution range and center area. In the Sixth Assessment Report of the IPCC, the possible development path of the global climate in the future was refined based on the SSP [36], and different possible change scenarios of greenhouse gas emissions were discussed. These scenarios were SSP1-1.9 and SSP1-2.6 with aggressive emissions reductions; SSP2-4.5 and SSP3-7.0 with moderate to high emissions; and SSP5-8.5 with extremely high emissions. With reference to the Bio-ORACLE integrated data [37], the 2050s and 2090s were selected as the two target time periods, and predictions were conducted under three different scenarios: SSP1-2.6, SSP2-4.5, and SSP5-8.5. The average of the 10 years from 2010 to 2020 was used as a reference to represent the current distribution, and the difference between the future distribution and the current distribution was compared by calculating the difference between the future probability and the current probability of the two squid species occurring together in each predicted grid point. The distribution and change trend of the two squid species together under the background of climate change were then quantitatively analyzed.

### 2.7. Statistical Analysis

One-way analysis of variance (ANOVA) was used to determine the statistical differences between predicted and observed values at all stations and during all seasons produced by the data-pool and prediction-pool methods, as well as differences among seasons, using the software WPS Office 3.0.2 (Kingsoft Office Corporation, Guangdong, China).

## 3. Results

### 3.1. Seasonal and Spatial Distribution Characteristics

**Figure 2:**
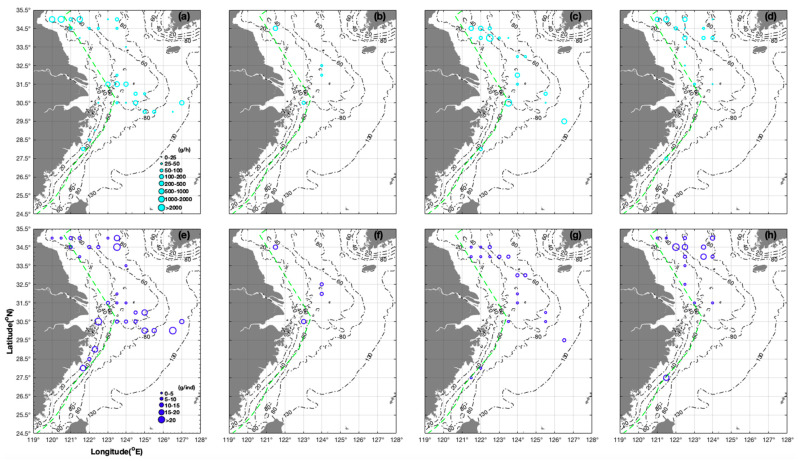
Seasonal distribution characteristics of CPUE_w_ (unit: g/h) shown in cyan (grouped as 0–25, 25–50, 50–100, 100–200, 200–500, 500–1000, 1000–2000, and >2000 g/h) and AIW (unit: g/ind) shown in blue (grouped as 0–5, 5–10, 10–15, 15–20, and >20 g/ind) for *Loliolus beka*. The size of the values is represented by the circles. The depth gradient (20–130 m) is represented by the black dash-dot line. (**a**–**d**) CPUE_w_ in (**a**) spring, (**b**) summer, (**c**) autumn, (**d**) winter; (**e**–**h**) AIW in (**e**) spring, (**f**) summer, (**g**) autumn, and (**h**) winter.

In terms of *L. beka*, the mean CPUE_w_ in all stations at 31.50° N was 166.68 g/h, compared with mean CPUE_w_ range of 59.50–68.09 g/h at 30.00° N, 30.50° N, and 31.00° N; AIW increased in the order of 2.75 → 3.54 → 12.93 → 11.80 → 17.98 g/ind corresponding to 32.00° → 31.50° → 31.00° → 30.50° → 30.00° N, indicating mainly smaller juveniles in the southern South Yellow Sea and larger juveniles in the northern East China Sea (Figure 2a,e). During the summer, AIW increased in the order of 0.80 → 2.06 → 3.02 → 5.87 → 5.90 g/ind, corresponding to 34.00° N 121.50° → 122.00° → 122.50° → 123.00° → 123.50° E (Figure 2g). Mean CPUE_w_ values were 1463.69 g/h and 1167.58 g/h, corresponding to all survey stations at 34.00° N and 30.50° N, respectively (Figure 2c). During the winter, CPUE_w_ and AIW ranged from 136.80 to 755.20 g/h and from 3.63 to 4.275 g/ind, respectively, at 35.00° N 121.00–121.50° E compared with 22.60–108.70 g/h and 9.05–11.30 g/ind, respectively, at 35.00° N 122.50–124.00° E (Figure 2d,h). The largest individuals (51.04 g/ind) were recorded at 34.50° N 122.00° E, whereas the smallest (2.00 g/ind) were recorded at 33.50° N 122.50° E, indicating the potential locations of nursery grounds (Figure 2h). AIW increased in the order of 2.00 → 3.40 → 4.40 → 16.60 g/ind, whereas this was 13.60 → 16.00 → 28.26 → 66.40 g/h for CPUE_w_, corresponding to all stations at 33.50° N → 32.50° N → 31.50° N → 27.50° N (Figure 2d,h).

**Figure 3:**
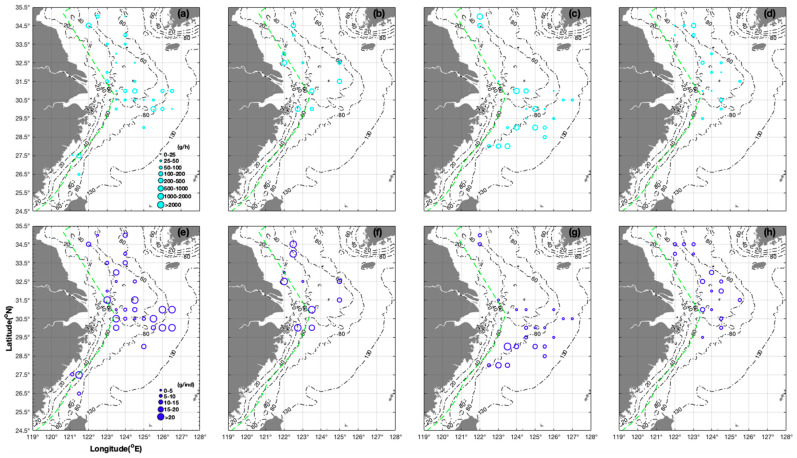
Seasonal distribution characteristics of CPUE_w_ (unit: g/h) shown in cyan (grouped as 0–25, 25–50, 50–100, 100–200, 200–500, 500–1000, 1000–2000, and >2000 g/h) and AIW (unit: g/ind) shown in blue (grouped as 0–5, 5–10, 10–15, 15–20, and >20 g/ind) of *Loliolus uyii*. (**a**–**d**) CPUEw in (**a**) spring, (**b**) summer, (**c**) autumn, (**d**) winter; (**e**–**h**) AIW in (**e**) spring, (**f**) summer, (**g**) autumn, and (**h**) winter.

In spring, the AIW of *L. uyii* ranged from 1.30 to 36.46 g/ind at 26.50°–35.00° N 121.13°–126.50° E (Figure 3e). During the summer, CPUE_w_ was 496.80 g/h and AIW was 20.70 g/ind at 32.50° N 122.00° E (Figure 3b,f); AIW ranged from 3.50 to 12.00 g/ind at 32.50° N 123.00°–125.00° E (Figure 3f). During the autumn, AIW ranged from 1.70 to 5.49 g/ind (mean of 3.75 g/ind) at 29.50°–31.50° N, and from 6.00 to 28.90 g/ind (mean of 13.01 g/ind) at 28.00°–29.00° N, indicating larger *L. uyii* north compared with south of 29.00° N (Figure 3g). AIW ranged from 2.30 to 3.58 g/ind at 31.00° N, from 1.70 to 4.40 g/ind at 30.50° N, and from 3.60 to 5.49 g/ind at 30.00° N, suggesting that most *L. uyii* larvae occurred in the north of the study region (Figure 3g). During winter, CPUE_w_ ranged from 9.84 to 101.50 g/h (mean of 43.11 g/h), whereas that of AIW was 2.50–12.33 g/ind (mean of 7.92 g/ind), at 29.50–34.50° N 122.00–125.50° E, showing that nursery grounds occurred in inshore areas (e.g., 18.20 g/h and 6.07 g/ind at 34.00° N 122.00° E compared with 63.60 g/h and 3.975 g/ind at 34.00° N 123.00° E) (Figure 3d,h).

In terms of mean CPUE_w_ at all stations, there was a seasonal order of autumn > spring > winter > summer for *L. beka*, and of autumn > spring + summer > winter for *L. uyii* (Table 1). Both species had high CPUE_w_ in autumn, with the CPUE_w_ and CPUE_n_ of *L. beka* being~three- and fivefold higher, respectively, compared with those of *L. uyii*. Meanwhile, the CPUE_w_ range of *L. beka* was largest during the autumn. The AIW of *L. beka* was 4.29 g/ind (range 0.80–8.00 g/ind, indicating both larvae and juveniles) during the autumn, with AIW values in other three seasons being similar 10.63 g/ind (range 1.24–40.88 g/ind) during the spring, 10.33 g/ind (range 6.50–14.40 g/ind) during the summer, and 11.29 g/ind (range 2.00–51.05 g/ind) during the winter), revealing that juveniles and adults occurred during the spring and winter, and only juveniles occurred during the summer (Table 1). The AIW of *L. uyii* was highest during the summer (19.54 g/ind of the parent groups), compared with 7.80 and 7.92 g/ind during the autumn and winter, respectively; the upper limit of AIW values occurred in the order of summer (40.03 g/ind) > spring (36.46 g/ind) > autumn (28.90 g/ind) > winter (12.34 g/ind), highlighting that spawning occurred from summer to autumn and nurseries from spring to winter (Table 1). Group sizes of 5–10 g/ind occurred in the order of spring (12 stations) > autumn (six stations) > summer and winter (two or three stations) for *L. beka*, compared with seven to nine stations during spring, autumn, and winter for *L. uyii* (Table 1).

### 3.2. Environmental Variables and Most Suitable Habitat Range

**Figure 4:**
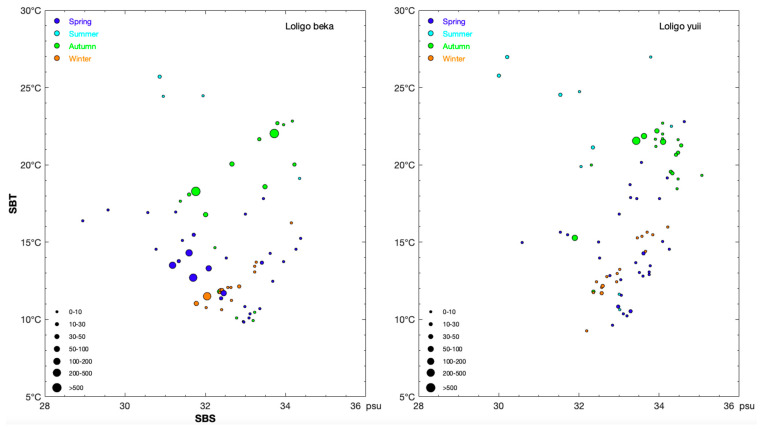
Relationship between sea bottom salinity (unit: psu) and sea bottom temperature (unit: °C) for CPUE_n_ sizes classified by group (0–10, 10–30, 30–50, 50–100, 100–200, 200–500, and >500 ind/h) of the species *Loliolus beka* (left panel) and *Loliolus uyii* (right panel). The data in spring, summer, autumn, and winter are denoted by blue, light sky blue, green, and brown-red solid circles, respectively.

*L. beka* showed narrow salinity (range SBS range: 28.95–34.38‰) and wide temperature (SBT range: 9.85–25.70 °C) ranges across the study year (Table 2). CPUE_n_ was 11.58–256.55 ind/h with an SBS range of 31.19–33.41‰ and a SBT range of 11.37–15.48 °C during the spring; there was a near-shore point with low SBS (30.86‰) and high SBT (25.70 °C) during the summer; the SBS (31.59–34.22‰) and SBT (16.79–22.70 °C) ranges during the autumn were wider compared with during the spring, and a high CPUE_n_ occurred at the station with low SBS (31.76‰) and SBT (18.29 °C) and at the station with high SBS (33.71‰) and SBT (22.02 °C) (Figure 4). *L. beka* inhabited areas with a narrower water temperature range (SBT: 11.03–12.15 °C) and a wider salinity range (SBS: 31.77–32.85‰) during the winter, representing the catches found in near-shore areas during this season (Figure 4). The lower limit of SBT of *L. beka* from the spring to autumn was lower than the lower limit of SST, occurring in the order of autumn (18.00 − 9.92 °C = 8.08 °C) > summer (25.76 − 19.13 °C = 6.63 °C) > spring (13.23 − 9.85 °C = 3.38 °C), whereas SST and SBT were similar during the winter (SST: 10.58–16.11 °C versus 10.62–16.26 °C) (Table 2). The upper limit range of SBS was 34.68–35.25‰, and its lower limit values occurred in the order of spring (28.95‰) > summer (29.39‰) > autumn (30.84‰) > winter (31.67‰) throughout the study area, suggesting that *L. beka* gradually move offshore from spring to winter (Table 2). The differences between the lower and upper limits of SST were 4.68 °C, 3.14 °C, 4.69 °C, and 5.53 °C during spring, summer, autumn, and winter, respectively (Table 2). The depth distribution of *L. beka* during spring and autumn ranged from 14.00 to 102.00 m, indicating a preference for coastal areas (15–60 m) during the summer and offshore areas (32.00–78.00 m) during the winter (Table 2). SSDO and SBDO were 4.92–7.20 mg/L and 3.55–4.30 mg/L, respectively, during the summer, potentially explaining the reduced number of *L. beka* during the summer (Table 2).

By contrast, *L. uyii* showed narrow salinity (SBS range: 32.98–33.61‰) and wider water temperature (SBT range: 10.52–14.27 °C) ranges during the spring; the SBT range increased to 21.14–26.96 °C during the summer with an SBS range of 30.00–32.34‰, representing a distribution adjacent to coastal areas during summer; during the autumn, *L. uyii* again showed a narrow salinity (SBS range: 33.44–34.55‰) and wider water temperature (SBT range: 19.46–22.20 °C), with a single point recorded for SBS (31.90‰) and SBT (15.27 °C). SBT (11.70–12.17 °C) and SBS (32.57–32.59‰) overlapped during the spring and winter, indicating that the occurrence of the life history stages of *L. uyii* can be separated into the period of winter to spring and summer to autumn (Figure 4). SBS and SBT ranged from 30.00 to 35.07‰ and from 9.26 to 26.97 °C, respectively, with lower (9.26–11.79 °C) and upper (15.97–26.97 °C) limit ranges of SBT across the year (Table 2). The ranges in SSS and SBS were similar during the spring (SSS: 30.58–34.41 °C versus SBS: 30.58–34.62 °C) and winter (SSS: 32.08–34.18 °C versus SBS: 32.19–34.22 °C), whereas there was a wider SBS than SSS range during the summer (SBS: 30.00–34.30‰ versus SSS: 29.81–33.20‰) and autumn (SBS: 31.90–35.07‰ versus SSS: 31.87–34.12‰) (Table 2). Depth range was similar during the summer and winter (16.00–68.00 m versus 19.00–70.00 m, respectively) (Table 2). SBDO was a little lower compared with SSDO during the summer (SSDO: 4.51–6.97 mg/L versus SBDO: 3.56–6.47) (Table 2).

### 3.3. Environment Variable Filtering, Response Curves, and Merged Spatial Distribution of Models

We detected a higher *p*-value for the data-pool method (t = 0.6827, *p* = 0.4951) and prediction-pool method (t = 0.1992, *p* = 0.8422), and significant differences among observed values and prediction values among different seasons. According to AIC values and the sum of plausibility, we selected key explanatory variables in the sdmTMB models of *L. beka*, *L. uyii* and the data-pool method (see Table 3). Depth was the explanatory variable, and the AIC value was the lowest at 367.66 in the model of a single explanatory variable of *L. uyii*. Similarly, for the species *L. beka*, AIC value was the lowest in 352.95 when SSS was regarded as the only explanatory variable (Table 3). In the condition of not adding any explanatory variables and introducing spatial random effects, the AIC value could be arrived at 559.22 in the data-pool model; SST was included as the most explanatory variable and the AIC value was the least (509.34) in a single explanatory variable model (Table 3).

**Figure 5:**
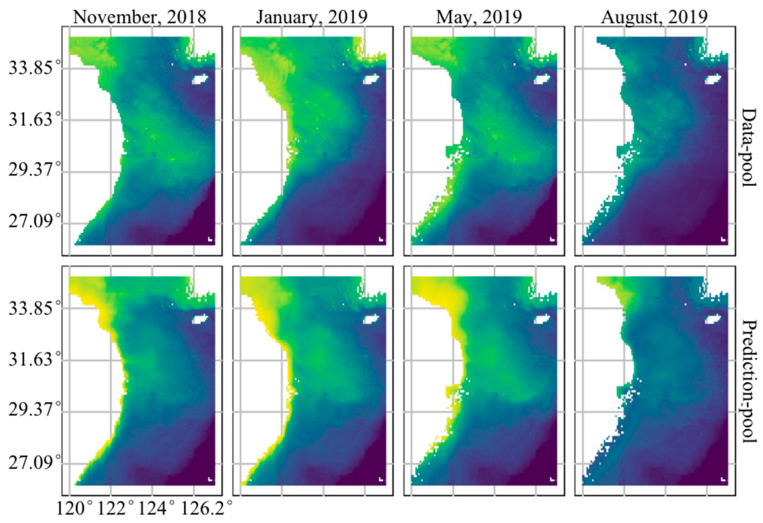
Seasonal distributions of both squid species in the East China and southern Yellow Seas predicted by the data-pool and prediction-pool methods. The horizontal and vertical coordinates are the UTM coordinate system, where the *x* axis represents the longitude, the *y* axis represents the latitude, and the color represents the joint probability of occurrence of both species. Blue and black represent lower values, and yellow and green represent higher values.

Based on the selected optimal models, we predicted the spatial distribution of *L. beka* and *L. uyii* in different seasons using the data-pool and prediction-pool methods (Figure 5). The predicted high-value areas, with an occurrence possibility > 50% of the overall spatial distribution of the two species, were similar, with similar patterns in different seasons. Both methods indicated that the high-value area of both species included the coastal areas of the southern Yellow Sea and the central East China Sea in autumn, similar to the situation during the spring, which included an enlarged area encompassing from shallow coastal areas to the central areas of survey region; although there were variations in the spatial distributions of high-value areas during the winter, the core distribution area of *Loliolus* spp. included the coastal areas of the southern Yellow Sea and the northern areas from the central areas of the East China Sea in winter; high-value areas decreased significantly during the summer (Figure 5).

### 3.4. Future Scenario Forecasting

**Figure 6:**
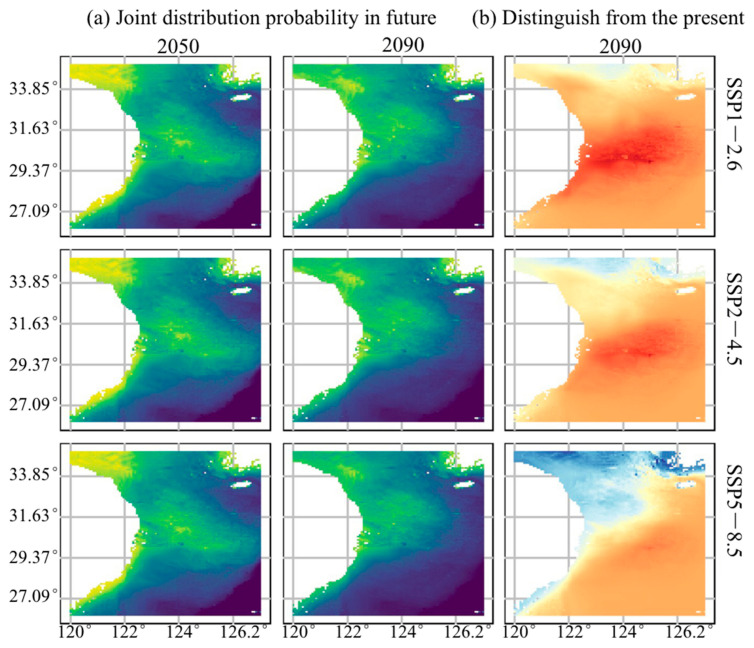
Changes in the joint spatial distribution of both squid species under three climate warming scenarios. (**a**) Joint probability of squid distribution during the 2050s and 2090s under different climate scenarios. Blue and black represent lower values, and yellow and green represent higher values. (**b**) Differences between the predicted distribution during the 2090s and the current distribution probability. Blue represents low values, and yellow and orange-red represent median values, and red represents high values.

We used the data-pool model to predict the spatial distribution variations of *L. beka* and *L. uyii* in a whole year under three future climate warming scenarios: SSP1-2.6, SSP2-4.5, and SSP5-8.5 (Figure 6a). The prediction results showed that, in all scenarios, by the 2050s, the joint distribution areas of *L. beka* and *L. uyii* would have gradually expanded, with more high-value distribution areas in the central East China Sea and southern Yellow Sea. However, by the 2090s, the distribution area of both species in survey locations would have significantly reduced in the coastal areas of the southern Yellow Sea and middle areas of the East China Sea compared with the case of the 2050s, with decreased strengths of high-value distribution areas. By comparing the case of the 2090s with the current predicted average values in the whole year as described in Section 3.3, we identified the areas of decreasing occurrence possibility (described in cold colors) and potential increasing occurrence (described in warm colors) (Figure 6b). In the case of the SSP1-2.6 scenario with an active carbon reduction strategy, there was a slight effect on the current distribution of both species, with a slightly decreasing occurring possibilities for the distribution area of southern Yellow Sea and a slightly increasing for the deep areas of the central East China Sea. In the SSP2-4.5 scenario, with moderate to higher CO_2_ emission levels, the distribution area of both species further decreased in the southern Yellow Sea, whereas there was a possibility that some areas of the central East China Sea still showed a potential increase. In the case of the SSP5-8.5 scenario with high-emission, the distribution areas of both species generally showed a downward trend across the whole survey area, being more significant in the northern area, and also in the coastal areas to a certain extent (Figure 6b).

## 4. Discussion

### 4.1. Models Evaluation and Predictive Effects of Data-Pool Model

By comparing the index of the AUC value, Brier score, and Matthews coefficients (MCC value), we quantitatively evaluated the prediction results of the methods including prediction-pool and data-pool (Table 4). The AUC value evaluating classification accuracy of data-pool model was better than that of prediction-pool (0.8597 against 0.7741); the Brier score evaluating the prediction precision of data-pool model was lower than that of the prediction-pool (0.1292 against 0.1580); the MCC value evaluating bipartite model performance of data-pool model was approaching to 1.00 closer than that of prediction-pool (0.3879 against 0.2402); thus, the prediction model using data-pool method was significantly better than the model using prediction-pool method after analyzing the above three indices (Table 4). In this study, we explored the impacts of data merging for *Loliolus* spp. on the created models in the framework of the sdmTMB model. Thus, we concluded that it was possible to use the data-pool method to accurately describe the distribution mode of *L. beka* and *L. uyii* by combining data of *Loliolus* spp. species *L. beka* and *L. uyii* under the framework of the sdmTMB model, which can bring benefits to the studies of habitat distribution assessments and predictions in the case of closely related species that cannot be distinguished in fisheries catch data.

In our study, the method of data-pool can expand the scale of the data set to obtain better data support and improve the accuracy of parameter estimation in the case of the model fitting of spatiotemporal distributions from the limited data sets of the two squid species, especially for the sdmTMB model with its complex structure. The introductions of spatial and spatiotemporal effects greatly improve the explanatory ability of the created models [38]. By contrast, although the data set of a single species can better show the ecological niche characteristics of that species, it is difficult to estimate the model parameters and fully capture such complex reactions because of the limited amount of data available.

Finally, various factors decrease the prediction accuracy of the models using the data-pool method, such as the quantity and quality of the data set, and the predictability and regularity of the target species distribution [39]. Particularly if there is obvious ecological niche differentiation of closely related *Loliolus* spp., the responses to the same environmental factors are significantly different, leading to the breakup of model fitting of the data-pool method. The addition of spatial random effects into the sdmTMB model structure enhances the prediction performance of the created model [40]. Moreover, the selected environmental variables, regardless of whether they can precisely represent the ecological needs and distribution patterns of the target species, as well as the spatial and temporal resolution, and the representativeness of variables, all directly impact the predictive ability of the created model [41]. Fishing and interactions among species can also cause immediate and lasting changes in species distributions [42]. Therefore, we need to evaluate the specific performance of the created models under different ecological scenarios based on the standard of the data attributes of the target species, the effects of environmental variables, and their ecological niche characteristics, to further understand the actual effects of different prediction methods and to promote the full utilization of limited data sets.

### 4.2. The Life History Characteristics and Seasonal–Spatial Distribution

Shen et al. (2010) used fishery-independent survey data from 2008 to 2009 at 26.00°–35.00° N 121.00°–126.50° E to identify the seasonal order in CPUE_w_ for *L. beka* as follows: autumn (1114.90 g/h in November) > winter (274.40 g/h in February) > summer (4.60 g/h in August); occurrence frequency: autumn (48.30%) > winter (29.20%) > summer (3.40%) > spring (1.70% in May) [3]. In addition, they argued that individuals in offshore areas were larger than those in coastal areas; and more individuals were found in the northern and central survey areas compared with the southern survey areas [3]. Yang et al. (2018) also found higher CPUE_w_ in coastal shallow areas compared with deep sea areas [43]. Jin et al. (2020) argued that *Loliolus* spp. showed higher abundances in coastal areas and preferred to stay in warmer inshore areas [44]. In our study, AIW of 2.81 g/ind and 19.02 g/ind occurred at a CPUE_w_ range of 2.90–60.11 g/h at 35.00° N 123.00°–123.50° E during the spring, representing juveniles with high CPUE_w_ in inshore areas (60.11 g/h) and dispersal feeding of juveniles in offshore areas (Figure 2a,e). For *L. uyii*, the mean CPUE_w_ increased in the order of 16.24 → 28.80 → 62.13 → 71.84 → 102.50 g/h corresponding to 32.50° N → 32.00° N → 31.50° N → 31.00° N → 30.00° N, with AIW of 1.30–5.20 g/ind at 32.50° N and 4.80 g/ind at 32.00° N 123.00° E, indicating that most *L. uyii* were juveniles (Figure 3a,e). We suggest that the seasonal variations in the distribution of these two species result from their short life cycle, rapid growth, and seasonal migration characteristics.

Second, Shen et al. (2010) reported a higher CPUE_w_ of *L. beka* at 34.00° N 123.00° E during the spring, at 31.50° N 124.50° E during the autumn, and at 35.00° N 123.00° E during the winter [3]. Chen et al. (2020) reported higher CPUE_w_ in northern Zhoushan (30.50° N), Taizhou coastal areas near 28.50° N, offshore areas of Nanji Islands (27.00°–27.50° N 121.00° E), and offshore areas of Taizhou Islands (122.00° E) [5]. Jin et al. (2004) revealed that *Loliolus* spp. overwinter in the central Yellow Sea from December to March, and then migrated to the coastal Yellow Sea for feeding and spawning during late spring and summer, before migrating back to the overwintering ground during late autumn and winter, with an optimal SST of 18.6–20.9 °C and CHLA of 2.30–3.76 mg/m^3^ [45]. In our study, *L. uyii* individuals recorded during the spring were smaller in inshore areas and larger in offshore areas (e.g., 1.30 g/ind at 32.50° N 123.50° E versus 5.20 g/ind at 32.50° N 124.50° E), and AIW increased in the order of 4.00 → 9.55 → 13.50 → 28.80 → 34.20 g/ind, corresponding to 31.00° N 123.50° → 124.00° → 124.50° → 126.00° → 126.50° E, indicating potential nursery locations in coastal areas with low AIW (Figure 3a,e).

Fan et al. (2017) reported that *Loliolus edulis* demonstrates reproductive migration to the north of the East China Sea during the spring to summer, and then migrates to the south during the autumn to winter [46]. In our study, both species shifted to shallow inshore areas, especially in the southern Yellow Sea and northern East China Sea; CPUE_w_ increased with SBT in spring, when both species spread back out into the central areas of the survey area, with most being adults, correlating with increasing AIW and CPUE_w_ during the summer and indicating a reproductive season at the turn of summer into autumn, when they migrated from offshore to coastal areas. In the latter, the highest CPUE_w_ occurred in juveniles during the autumn, indicating the in situ transformation of spawning grounds in the summer to nursery grounds in the autumn.

In this paper, we hypothesized that individuals of both species remaining in the northern survey area might migrate to the northern Yellow Sea and Bohai Sea to spawn, whereas individuals of both species remaining in the central and southern Yellow Sea might migrate to coastal areas to spawn; Both species will have finished breeding by the autumn, at which point new-born larvae in the north of the survey area would migrate to the southern Yellow Sea, whereas new-born larvae in middle and southern areas of the survey will gradually migrate to offshore areas, resulting in a distribution ranging from coastal sea areas to the central survey area, similar to the distribution seen during the spring. We suggest that the central part of the survey area provides a suitable SBT and SBS and favorable hydrodynamic conditions during the spring and autumn, which is why both species migrate to this region during these seasons. Studies of the migration routes of two species in this study in the East China Sea region are limited and, thus our hypothesis requires verification to also reveal more details about the population dynamics of these two species.

### 4.3. Fisheries Management Strategies to Address Impacts of Climate Change on Species Distributions

Through analyzing the spatial distribution in a whole year of these two *Loliolus* spp. species under three predictable climate scenarios (SSP1-2.6, SSP2-4.5, and SSP5-8.5), we found that climate warming asserted a significant impact on their spatial distribution. Climate warming can cause changes in the latitude and depth of single-species and multi-species distribution, as well as the habitat range, and migration routes, and thus significantly impacting the utilization of fishery resources and biodiversity conservation [47,48]. Our research into the seasonal–spatial distributions of two species showed that the high-value areas and peak values of both species distribution would change by the 2050s, with the spatial distribution range of both species increasing in the medium and long term. Previous work also showed that, under global warming, the habitat spatial distribution range of cephalopods would expand in the short term [48]. This expansion might be due to the future range of the increased SST remaining within the tolerance range in current areas of high CPUE_w_ for both species; furthermore, with the increasing SST, the metabolic rate of *Loliolus* spp. species might accelerate, promoting their growth and reproductive rate [49]; in this instance, some previously unsuitable areas with lower water temperatures would become suitable for both species in the short term. However, by the 2090s, the spatial distribution ranges of both species would shrink compared with the 2050s, with high-value distribution areas and peak values reduced in coastal areas and the central East China Sea. Under different climate warming scenarios, comparison of the projected future distributions with the current situation, with increases in carbon emissions and rising SST, the concentration and peak values of the spatial distribution of both species would continue to shrink, especially in the northern study area, with a slight decline in the coastal areas. Under the SSP1-2.6 and the SSP2-4.5 scenarios, some areas of the central East China Sea showed the potential to increase the possible occurrence of both species. We believe that our study follows the direction given by Rodhouse et al. (2014) concerning the simultaneous impact of various environmental factors on the changes in the squid population dynamics [50]. Previous studies highlighted that, in the context of global climate change, marine organisms would migrate to higher latitudes [51,52] in search of more suitable SST to decrease their survival pressures. Accordingly, both species stocks in the northern part of the survey area were assumed to move to the north-central Yellow Sea as new more suitable habitats. Both species stocks in the central and southern survey areas were assumed to move to the middle areas of the East China Sea as potential new habitats. Considering the future possible variations of both species populations, the fisheries managers should consider scientific adaptive management strategies, including the implementation of sustainable fishing policies, the protection of key habitats, and the enhancement of monitoring and assessments of marine ecosystem health to identify the potential impacts of climate change on living marine organisms. Through improved predictions of the habitat distribution of target species, the results of this study can bring benefits to the long-term sustainable utilization of both species, promote the overall health and stability of the marine ecosystem, and the continuous implementation of fisheries management actions.

## 5. Conclusions

Based on our results, the prediction model using the data-pool method was significantly more representative than the model using the prediction-pool method. Thus, the data-pool method was able to accurately describe the distribution of *L. beka* and *L. uyii* based on their combined data under the framework of the sdmTMB model.

(1) The mean CPUE_w_ at 31.50° N was more than that at 30.00° N, 30.50° N, and 31.00° N, with increasing individual size from 32.00° N to 30.00° N, indicating that there were mainly younger juveniles in the southern Yellow Sea and larger juveniles in the northern East China Sea during this season. Higher CPUE_w_ and smaller AIW were recorded in inshore areas and vice versa in offshore areas during the autumn and winter. AIW and CPUE_w_ expanded from 33.50° N to 27.50° N during the winter. By contrast, the mean CPUE_w_ for *L. uyii* increased from 32.50° N to 30.00° N during the spring, with individual size expanding from inshore to offshore areas at 31.00° N; the individual size was also greater in inshore area compared with offshore areas at 32.50° N during the spring and summer, whereas individuals were smaller to the north of 29.00° N and larger to the south of 29.00° N during the autumn. In terms of mean CPUE_w_ at all sample sites, there was a seasonal order of autumn > spring > winter > summer for *L. beka*, and of autumn > spring plus summer > winter for *L. uyii*.

(2) The high-value areas of both species included inshore areas of the southern Yellow Sea and mid-East China Sea during the autumn. The core distribution area of both *Loliolus* spp. included inshore areas of the southern Yellow Sea and northern areas, having moved from the central areas of the East China Sea during the winter.

(3) The models predicted that, by the 2050s, the joint distribution areas of both species will have gradually expanded, with more high-value distribution areas in the central East China Sea and southern Yellow Sea. However, by the 2090s, the distribution area of both species in the survey locations would have significantly reduced in the coastal areas of the southern Yellow Sea and middle areas of East China Sea compared with during the 2050s.

## Figures and Tables

**Table 1:** Seasonal data for catch per unit effort by weight (CPUE_w_) and by number (CPUE_n_) data and average individual weight (AIW) for *Loliolus beka* and *Loliolus uyii*.

	*Loliolus beka*				*Loliolus uyii*			
	Spring	Summer	Autumn	Winter	Spring	Summer	Autumn	Winter
Mean CPUE_w_ at all stations	28.03	1.95	84.65	14.03	12.51	10.43	26.74	6.21
Mean CPUE_w_ at collection stations	136.31	68.34	597.25	103.85	58.79	146.01	161.70	43.11
Value range of CPUE_w_	2.90–1042.76	29.16–147.60	4.80–7196.00	13.60–755.20	8.00–277.20	45.50–496.80	10.47–809.41	9.84–101.50
Mean CPUE_n_ at all stations	6.37	0.18	26.12	2.91	1.07	0.70	4.79	0.89
Mean CPUE_n_ at collection stations	30.95	6.40	184.27	21.52	5.05	9.79	29.00	6.15
value range of CPUE_n_	1.00–256.55	3.60–12.00	1.50–2380.00	1.00–208.00	1.00–21.05	2.00–24.00	1.50–225.88	1.97–16.00
Mean AIW	10.63	10.33	4.29	11.29	14.95	19.54	7.80	7.92
Value range of AIW	1.24–40.88	6.50–14.40	0.80–8.00	2.00–51.05	1.30–36.46	3.50–40.03	1.70–28.90	2.50–12.34
Groups classified by AIW	Station number							
0–5	7		12	6	4	2	9	4
5–10	12	2	6	3	9		7	7
10–15	2	2		2	6	2	3	5
15–20	5			3	2	1	1	
>20	3			1	9	5	1	

**Table 2:** Seasonal in situ ranges of environmental factors in the study area ^a^.

Factor	Spring	Summer	Autumn	Winter
*Loliolus beka*				
Depth (m)	19.00–101.00	15.00–60.00	14.00–102.00	32.00–78.00
SST (°C)	13.23–17.91	25.76–28.90	18.00–22.69	10.58–16.11
SBT (°C)	9.85–17.83	19.13–25.70	9.92–22.83	10.62–16.26
SSS (‰)	28.80–33.50	29.88–31.93	30.49–33.71	31.50–34.06
SBS (‰)	28.95–34.38	30.86–34.35	31.37–34.22	31.77–34.14
SSDO (mg/L)	7.84–8.61	4.92–7.20		8.00–8.84
SBDO (mg/L)	7.76–9.18	3.55–4.30		7.97–8.55
*Loliolus uyii*				
Depth (m)	35.00–90.00	16.00–68.00	35.00–107.00	19.00–70.00
SST (°C)	13.17–24.81	25.03–29.26	18.97–23.22	9.14–15.94
SBT (°C)	9.64–22.79	10.64–26.97	11.79–22.70	9.26–15.97
SSS (‰)	30.58–34.41	29.81–33.20	31.87–34.12	32.08–34.18
SBS (‰)	30.58–34.62	30.00–34.30	31.90–35.07	32.19–34.22
SSDO (mg/L)	7.84–8.57	4.51–6.97		8.02–8.76
SBDO (mg/L)	7.76–9.23	3.56–6.47		8.01–8.68

^a^ Abbreviations: SST, sea surface temperature; SBT, sea bottom temperature; SSS, sea surface salinity; SBS, sea bottom salinity; SSDO, sea surface dissolved oxygen; SBDO, sea bottom dissolved oxygen.

**Table 3:** Selection of explanatory variables for the model of *Loliolus uyii*, *Loliolus beka*, and data-pool method.

Category	Formula (Fixed Effects)	Random Effects ^a^	AIC	Sum of Log-Likelihoods ^b^
*Loliolus uyii*	y ~ 1 ^c^	off	370.70	-
	y ~ 1	on	370.47	−185.85
	y ~ s(Depth) ^d^	on	367.66	−183.91
	y ~ s(Depth) + s(SST)	on	370.25	−185.20
	y ~ s(Depth) + s(SST) + s(SSS)	on	-	-
*Loliolus* *beka*	y ~ 1	off	394.14	-
	y ~ 1	on	351.74	−214.12
	y ~ s(SSS)	on	352.95	−170.47
	y ~ s(Depth) + s(SSS)	on	-	-
	y ~ s(Depth) + s(SSS) + s(SST)	on	326.32	−160.38
Data-pool	y ~ 1	off	559.22	-
	y ~ 1	on	524.04	−299.67
	y ~ s(Depth)	on	517.49	−258.47
	y ~ s(SST)	on	509.34	−269.97
	y ~ s(SSS)	on	527.77	−262.01
	y ~ s(SST) + s(SSS)	on	507.52	−215.41
	y ~ s(Depth) + s(SST)	on	493.47	−187.55
	y ~ s(Depth) + s(SSS)	on	-	-
	y ~ s(SSS) + s(SST)	on	500.65	−215.41
	y ~ s(Depth) + s(SSS) + s(SST)	on	-	-

^a^ On and off: consideration of spatial random effects or not, respectively. ^b^ Indicates the mean of the total log-likelihood after conducting 100 cross-validations of the model; ^c^ ‘1’ indicates the inclusion of an intercept only, with no explanatory variables; ^d^ s(XXX), where XXX is a parameter: using natural spline smoothing.

**Table 4:** Evaluation of the predictive performance of the data-pool and prediction-pool methods.

Method	Evaluation Index		
	AUC	Brier Score	Matthews Correlation Coefficient
Prediction-pool	0.7741	0.1580	0.2402
Data-pool	0.8597	0.1292	0.3879

## Data Availability

Data are contained within the article.
